# The effect of extended hemodialysis on nutritional parameters: a systematic review

**DOI:** 10.1007/s40620-022-01395-w

**Published:** 2022-08-12

**Authors:** Alireza Majlessi, James O. Burton, Daniel S. March

**Affiliations:** 1 Department of Cardiovascular Sciences, University of Leicester, Leicester, UK; 2 John Walls Renal Unit, University Hospitals of Leicester NHS Trust, Leicester, UK

**Keywords:** Extended haemodialysis, Dialysis, Body mass index, Nutrition, Systematic review

## Abstract

**Objective:**

This systematic review provides an up-to-date synthesis on the effects of extended hemodialysis on nutritional outcomes.

**Design and Methods:**

Ten databases were searched. Inclusion criteria were: randomised and non-randomised studies of extended hemodialysis (defined by > 15 h/week) with a comparator group which received conventional in-centre hemodialysis (usually ≤ 12 h per week). Outcomes of interest included lean body mass, protein and carbohydrate intake, body mass index, dry lean mass, water-soluble vitamin levels, serum levels of appetite hormones, and nutritional status as assessed by the PEW and SGA scoring tools.

**Results:**

Five studies were eligible. All investigated extended nocturnal hemodialysis (one with the addition of short daily), three were in-centre and two were at home. Range of duration for the included studies was 2–18 months. These studies reported data on lean body mass, protein and carbohydrate intake, body mass index, dry lean mass and water-soluble vitamin levels. There was insufficient homogeneity between the studies to meta-analyse the data. Extended hemodialysis had no significant effects on any of the reported outcomes except for lean body mass, where a significant increase was found, and water-soluble vitamin levels, where deficiency was identified in one of the included studies.

**Conclusion:**

There is currently no evidence to suggest that extended hemodialysis modalities impact nutritional parameters, although the quality of the available evidence is low.

**Graphical abstract:**

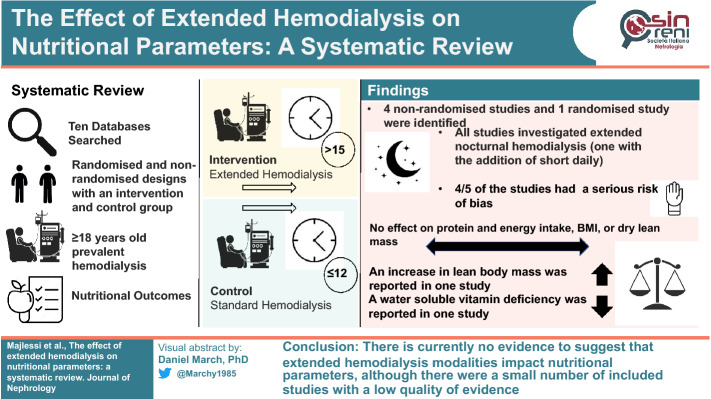

## Introduction

Individuals with end-stage kidney disease (ESKD) whom receive hemodialysis have a high mortality rate, standing at 20% within 1 year, and just under 50% at 5 years [1]. Nutritional status of hemodialysis patients has been associated with increased mortality [2], and increased morbidity [3], and therefore, finding ways to identify and correct malnutrition and in turn improve the nutritional status of hemodialysis patients is critical.

One such intervention that may be efficacious in improving the nutritional status of individuals receiving hemodialysis is extended hemodialysis (EHD). The existing evidence regarding EHD modalities, while limited, may show the potential to improve nutritional status when compared to conventional thrice-weekly hemodialysis. EHD has shown potential in preserving nutritional status in patients [4]. It is thought that the increased clearances associated with EHD can lead to a less restricted diet, with reduced need for phosphate binders [5]. This may lead to greater appetite and improved nutrition, which is associated with improved health-related quality of life and reduced mortality [6, 7]. Conversely, EHD may have some negative effects with some evidence that it can lead to significant amino acid losses [8], which can be detrimental to the nutritional status of the patients. Furthermore, hemodialysis itself is known to be one of the causes of inflammation in this population [9, 10], and may lead to reduced appetite and worsening protein-energy wasting [11].

Therefore, the primary aim of this systematic review is to provide an up-to-date assessment of the efficacy of EHD compared to standard dialysis therapy on the following nutritional markers: body mass index (BMI), lean body mass (LBM), dry lean mass, water soluble vitamins, protein and energy intake, circulating markers of appetite (i.e. Leptin, Ghrelin, Peptide YY), and nutritional status as assessed by the Subjective Global Assessment (SGA) and Protein Energy Wasting (PEW) scores.

## Methods

### Protocol registration

Methods of analysis and inclusion criteria were specified in advance and documented in a protocol that was registered on PROSPERO (International Prospective Register of Systematic Reviews) with the identifier CRD42021236356. This systematic review has been reported in line with the Preferred Reporting Items for Systematic Reviews and Meta Analyses (PRISMA) 2020 checklist [12].

### Settings and study population

#### Participants

Participants were required to be ≥ 18 years old and prevalent hemodialysis patients (receiving for > 3 months). Paediatric or pregnant individuals were excluded as were those receiving treatment in an acute setting.

#### Intervention

Participants with ESKD receiving extended-hours hemodialysis or hemodiafiltration. For the purpose of this review, extended-hours of hemodialysis or hemodiafiltration has been defined as any regimen that exceeds 15 h per week compared to standard, which was defined as ≤ 12 h across three sessions per week. The extended-hours hemodialysis or hemodiafiltration could be achieved through either long nocturnal, long day, or short frequent day hemodialysis either in-centre, satellite, shared care or in the home setting. The standard therapy had to be delivered in-centre.

#### Comparison

The comparator group will be patients receiving usual care, which is normally daytime in-centre hemodialysis taking place thrice weekly, usually with each session lasting for 3–4 h.

#### Outcomes

The outcomes included body mass index, lean body mass, dry lean mass, water soluble vitamins, protein and energy intake, circulating markers of appetite (i.e. Leptin, Ghrelin, Peptide YY), and nutritional status as assessed by the SGA and PEW scores.

### Study design

Eligible studies included randomised, quasi-randomised and non-randomised designs. Studies were required to have an intervention and concurrent comparator group receiving usual care.

### Search strategy and information sources

Searches were conducted to identify any relevant completed or ongoing systematic reviews using the following resources: Cochrane and PROSPERO National Health Service Centre for Reviews and Dissemination: Health Technology Assessment (HTA), and Database of Abstracts of Reviews of Effects (DARE). The following bibliographical databases and study registers were searched for complete and ongoing studies: MEDLINE, EMBASE, CINAHL, Cochrane Controlled Register of Trials (CENTRAL), ClinicalTrials.gov, the ISCRTN Registry, Conference Proceedings Citation Index (Web of Science™ Core Collection).

No limits were set regarding language, and all the databases were searched from conception to November 2021. Search terms used for MedLine have been provided (Appendix Table 4). Search results were compiled in Endnote (Clarivate Analytics, Philadelphia, PA). Duplicates were removed and a random selection of 10% of the total studies (*n* = 619) were screened for title and abstract independently by two reviewers (A.M & D.S.M) against the inclusion criteria. Agreement between the two reviewers was 99.5%, therefore the remainder of the studies (*n* = 5571) were screened (for title and abstract) by one reviewer (A.M). Reports not excluded based on title and abstract were retrieved and underwent non-blinded assessment by two reviewers (A.M & D.S.M).

### Selection criteria, data extraction, risk of bias and quality assessment

We developed, tested and refined a structured data collection form based on the Cochrane Data Extraction Template for Interventions. One reviewer (A.M) carried out data extraction for each paper, with a second reviewer (D.S.M) available for cross checking. For each individual study information was extracted on study methods, participants, intervention/comparison and outcomes.

### Study risk of bias assessment

Two reviewers (A.M, D.S.M) independently assessed the risk of bias within included studies using the “ROB-2” tool [13] for randomised controlled trials (RCTs) included. The overall risk of bias for each study was classified using the following criteria: low, some, or high risk of bias. For the included non-randomised studies, the “ROBINS-I” tool [14] was used. The overall risk of bias for each study was classified as either low, moderate, or serious risk of bias. Disagreements between the two reviewers were resolved through further discussion with a third reviewer (J.B).

### Analytical approach

Due to the insufficient amount of evidence (small number of heterogeneous studies with a range of different outcomes), we provide a narrative synthesis of our findings from the included studies as was pre-specified. This was structured around the effect of EHD on SGA and PEW score, BMI, protein and energy intake, LBM, water soluble vitamin levels and on appetite markers as pre-specified. Data for LBM was extracted for one study using Web-Plot Digitizer Version 4.5 [15]. Prior to conducting the systematic review, a pre-specified analytic plan was created for meta-analysis of study outcomes. This plan can be viewed in the study protocol, which is available to view on PROSPERO (CRD42021236356).

## Results

### Study selection

**Fig. 1:**
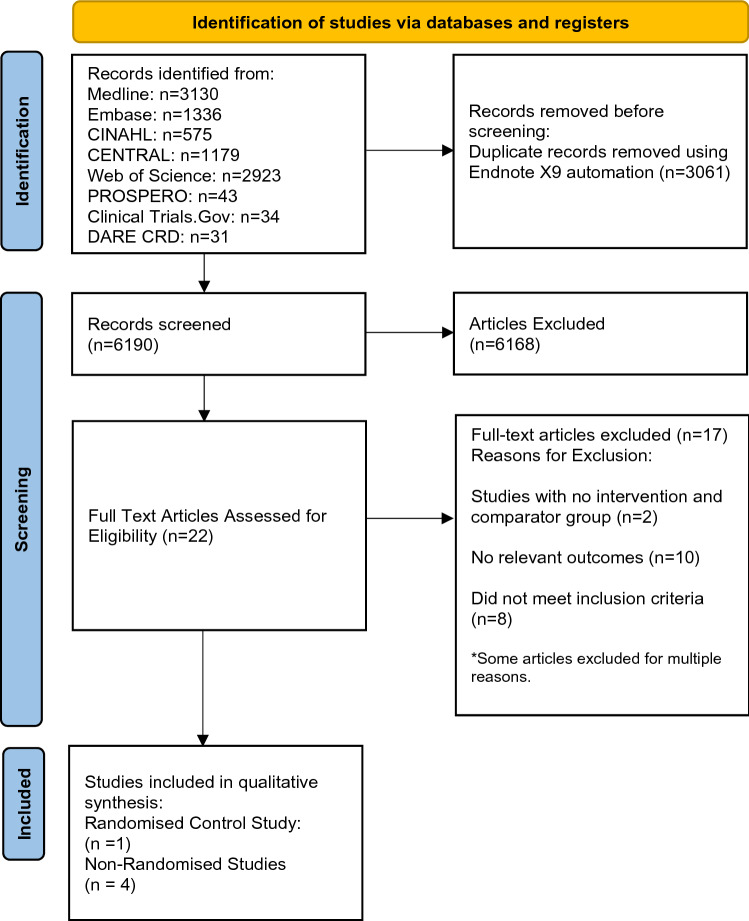
PRISMA flow diagram summarising search results

Figure 1 provides a flow diagram of study selection. Overall, 17 studies [16–32] were excluded at full text screening, with reasons given. Five studies were suitable for inclusion in this systematic review. These studies consisted of four non-randomised studies [33–36] and one RCT [37]. Table 1 represents a summary of the characteristics for all the included studies. These studies provided data on BMI, LBM, protein and carbohydrate intake, water soluble vitamin levels and dry lean mass. No included studies provided data for nutritional status as assessed by SGA/PEW scores or circulating markers of appetite.

**Table 1:** Summary of characteristics for each included study

Study	Country	Follow-up period	Participants	Age (Years), mean (± S.D.)	Dialysis vintage (months unless stated)	Ethnicity	Co-morbidities (number of participants with co-morbidity)	Study type (randomized/observational)	Details regarding intervention and control	Missing data (Reasons)	Hemodialysis or hemodiafiltration
Torigoe 2016	Japan	2 months	Intervention (*n* = 8) Usual care (*n* = 10)	Intervention: 44.5 (± 3.0) Usual Care: 68.1 (± 2.7)	Not Reported	Not reported	Not reported	Prospective observational	Intervention: 8 h three times a week hemodialysis sessions Control: 4–5 h three times a week hemodialysis sessions	Not reported	Hemodialysis
Schorr 2011	Canada	6 months	Intervention (n = 27) Usual Care (n = 25)	Intervention: 55.1 (± 12.4) Usual Care: 53.1 (± 13.4)	Mean dialysis vintage > 5 years	Intervention: 69% Caucasian ethnicity Control: 56% Caucasian ethnicity	Intervention: Diabetes mellitus (10) Ischaemic heart disease (10) Congestive heart failure (6) Peripheral vascular disease (4) Cerebrovascular disease (5) Control: Diabetes mellitus (11) Ischaemic heart disease (10) Congestive heart failure (5) Peripheral vascular disease (4) Cerebrovascular disease (3)	Randomised control trial	Intervention: 5–6 nights per week nocturnal hemodialysis sessions lasting 5–6 h Control: Conventional hemodialysis three times per week	14 Participants (Intervention Group) Reasons Not Reported 14 Participants (Control Group) Reasons Not Reported	Hemodialysis
Spanner 2003	Canada	18 months	Intervention (Daily HD: *n* = 11, Nocturnal HD: *n* = 12) Usual care (*n* = 22)	Intervention: SDHD: 45.3 (± 11.4) NHD: 44.2 (± 6.4) Usual Care: 48.8 (± 11.9)	Not reported	Not Reported	Daily HD: Diabetes (1) Hypertension (8) Heart disease (4) Hyperparathyroidism (6) Nocturnal HD: Diabetes (3) Hypertension (8) Heart disease (3) Hyperparathyroidism (3) Control Diabetes (4) Hypertension (8) Heart disease (3) Hyperparathyroidism (7)	Prospective Observational	Intervention: Nocturnal Hemodialysis Group: 5–6 times per week for 6–8 h while asleep + Short Daily Hemodialysis Group: 5–6 times per week for 1.5–2.5 h Control: Conventional Hemodialysis 3 times per week for 3.5–4.5 h	Intervention groups SDHD: 1–4 participants (depending on outcome measure) NHD: 6–7 participants (depending on outcome measure) No reasons reported Control group: CHD: 2–6 participants (depending on outcome measure) No reasons reported	Hemodialysis
Demirci 2013	Turkey	12 months	Intervention (n = 102) Usual care (n = 95)	Intervention: 47.1 (± 11.7) Usual care: 49.1 (± 11.9)	Intervention: 67 months (± 49) Control: 60 months (± 51)	Not Reported	Intervention: Diabetes (12) Cardiac disease (4) Erythropoietin use: 49% Anti-hypertensive drug use (28%) Phosphate binder use (79%) Control Group Diabetes (15) Cardiac disease (5) Erythropoietin use (60%) Antihypertensive drug use (33%) Phosphate binder use (84%)	Prospective Observational (Cohort Study)	Intervention: Three times/week nocturnal hemodialysis for 7–8 h per night Control: 3 times/week conventional hemodialysis for 3.5–4.5 h per night	45 Participants (Intervention Group) Transfer to another centre (*n* = 18) Discontinuation of therapy (*n* = 17) Did not attend second measurement (*n* = 5) Renal Transplantation (*n* = 4) Death (*n* = 1) 40 Participants (Control Group) Transfer to another centre (*n* = 27) Death (*n* = 5) Did not attend second measurement (*n* = 8)	Hemodialysis
Ipema 2014	Netherlands	12 months	Intervention (n = 21) Usual Care (n = 14)	MEDIAN (IQR) Intervention: 41 (36–51) Usual Care: 49 (36.5–66.5)	MEDIAN (IQR) Intervention 3 (1–6) years Control 3 (1–5) years	Intervention 10 Caucasian (91%) 1 Asian (9%) 0 Black Control 11 Caucasian (84% 1 Asian (8%) 1 Black (8%)	Intervention Diabetes Mellitus (1) Control Diabetes Mellitus (2)	Prospective Observational	Intervention: 8 h single-needle dialysis Nocturnal in-centre hemodialysis: Every other night Nocturnal home hemodialysis: 5–6 nights/week Control: 4 h three-time weekly double-needle dialysis	10 Participants (Intervention Group) Trouble Sleeping (*n* = 1) Sepsis (*n* = 1) Heart failure with hypotension (*n* = 4) Renal Transplantation (*n* = 1) Miscellaneous reasons (*n* = 3) 1 Participant (Control Group) Renal Transplantation	Hemodialysis

### Risk of bias and quality assessment

**Fig. 2:**
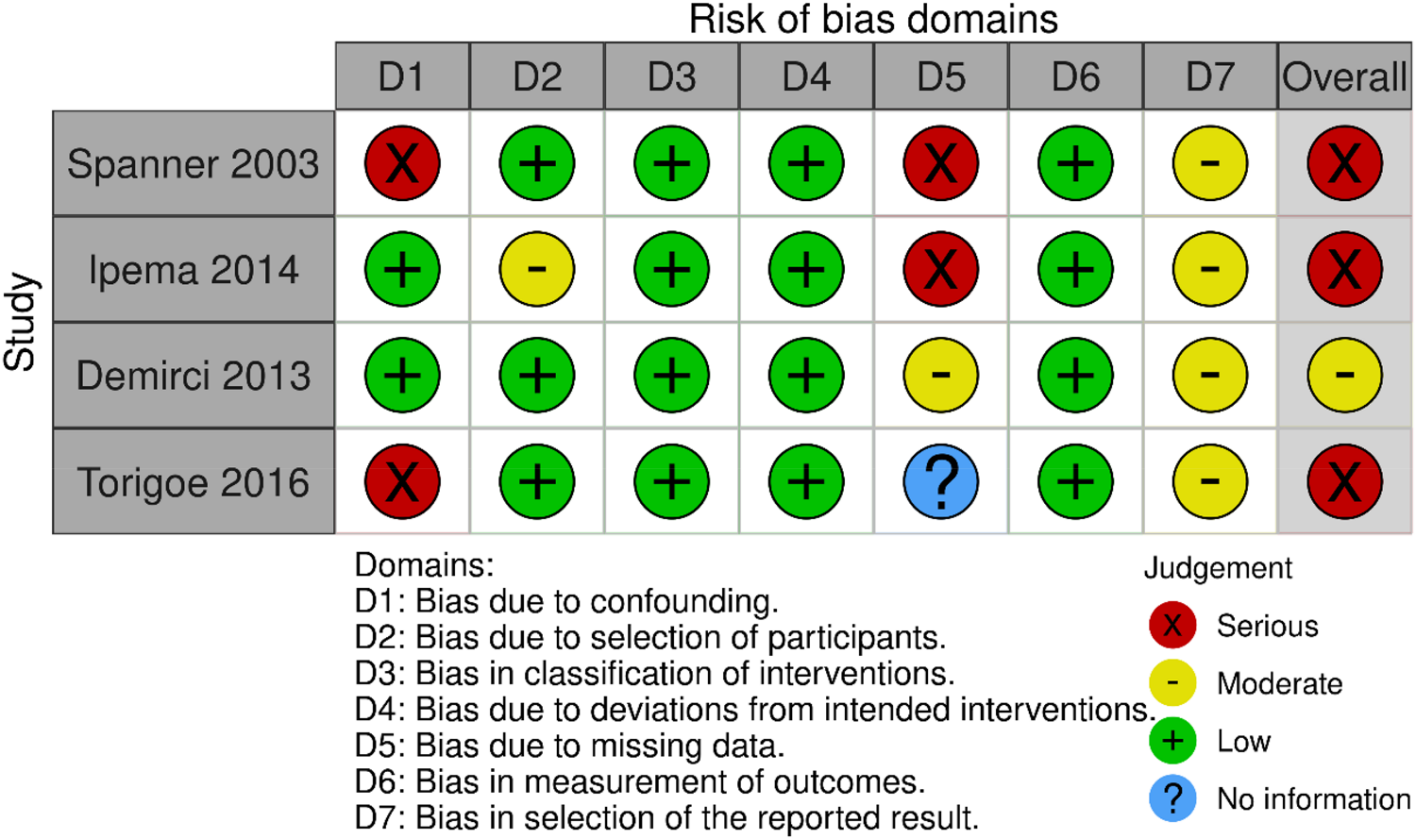
Risk of bias assessments for non-randomised trials using ROBINS-I tool

**Fig. 3:**
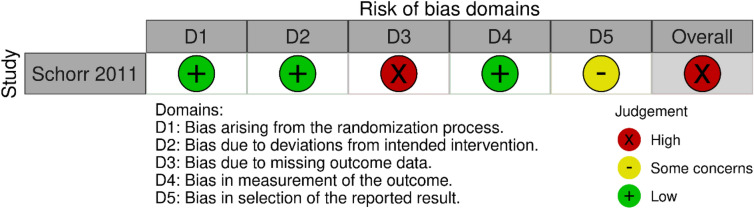
Risk of bias assessment for the included randomised control trial using ROB-2 tool

The Cochrane ROBINS-I tool [14] was used to assess the risk of bias for the four included non-randomised studies [33–36] (Fig. 2). The Cochrane ROB-2 tool [13] was used to assess risk of bias for the single included RCT [37] (Fig. 3). Overall, 4/5 studies [33–35, 37] were judged to be at serious risk of bias, while one study was graded to have a moderate risk of bias [36].

### Certainty assessment

A certainty assessment on each of the reported outcomes was carried out by using the “Grading of Recommendations, Assessment, Development and Evaluations” (GRADE) framework [38, 39] to subjectively assess the quality of the overall pool of evidence for each outcome. We followed expert guidance from a paper by Murad et al. [40] on how to apply the GRADE criteria when the results of a systematic review have been reported narratively. Overall, the certainty in the evidence (Table 2) retrieved from the included studies was rated as low for the following outcomes: BMI [33, 34, 37], LBM [33–35], protein and energy intake [37] and dry lean mass [36]. The certainty in the evidence available for water soluble vitamin levels [34] was rated as very low.

**Table 2:** GRADE certainty assessment results

Outcome	Effect	Number of participants	Certainty in the evidence
Body mass index	All the included studies reported no significant differences between extended hemodialysis and conventional hemodialysis with regard to BMI	131 Participants recruited (1 randomised control trial, 2 prospective observational studies)	LOW ⊕⊕OO (Due to serious risk of bias and imprecision)
Lean body mass	All the included studies reported no significant differences between extended hemodialysis and conventional hemodialysis with regard to lean body mass	98 Participants recruited (3 Prospective, observational studies)	LOW ⊕⊕OO (Due to serious risk of bias and imprecision)
Protein and energy intake	Only one included study, which reported no significant differences between extended hemodialysis and conventional hemodialysis with regard to protein and energy intake	51 Participants Recruited (1 Randomised Control Trial)	LOW ⊕⊕OO (Due to serious risk of bias, methodological issues, and imprecision)
Water soluble vitamin levels	Only one included study, which reported that water soluble vitamin levels were in the reference ranges for all three groups (NHD, SDHD, and CHD), except for vitamin C which was deficient amongst half of the NHD population. “Significant” differences were found in homocysteine levels, which were much lower in the NHD group when compared to the control group	45 participants Recruited (1 Prospective, observational study)	VERY LOW ⊕OOO (Due to serious risk of bias, serious methodological issues, and imprecision)
Dry lean mass	Only one included study, which reported that there was no significant adjusted mean difference in terms of dry lean mass between an extended hemodialysis group (NHD) and a conventional hemodialysis group	197 Participants Recruited (1 Prospective, cohort study)	LOW ⊕⊕OO (Due to moderate risk of bias and imprecision)

### Body mass index

Three of the included studies reported outcome information regarding BMI [33, 34, 37]. However, due to heterogeneity in study design (randomised and non-randomised design) it was not possible to perform a meta-analysis. Ipema 2014 [33] reported no significant difference between EHD and conventional hemodialysis (CHD) with regard to BMI, at twelve-month follow up (EHD group baseline: 24.7 ± 3.7 kg/m^2^ to 12 months; 24.8 ± 3.9 kg/m^2^; CHD group baseline: 25.8 ± 3.9 kg/m^2^ to 12 months; 25.8 ± 3.8 kg/m^2^). Schorr 2011 [37], the only RCT included in this systematic review, recruited an EHD group which carried out nocturnal hemodialysis 5–6 times per week, with each session lasting a minimum of 6 h. This study reported no significant difference between EHD and CHD with regard to their effects on BMI after six months (EHD group baseline: 27.3 ± 6.7 kg/m^2^ to six months; 27.4 ± 6.9 kg/m^2^; CHD group baseline: 23.5 ± 5.5 kg/m^2^ to six months; 23.4 ± 5 kg/m^2^). Spanner 2003 [34] did not state clearly when or how often BMI measures were taken, and the authors only state that there were “no significant changes in BMI for the 3 study groups” throughout the 18-month study period.

### Lean body mass

Three of the included studies provided outcome information regarding LBM [33–35]: Ipema 2014 [33] reported no significant change in LBM in either the EHD or the CHD group after 12 months (EHD group baseline: 49.9 ± 8.0 kg to 12 months; 48.6 ± 8.2 kg; CHD group baseline: 51.1 ± 7.8 kg to 12 months; 50.2 ± 7.2 kg). Spanner 2003 [34] did not report when or how frequently LBM was measured. The study showed no significant differences in LBM between the study groups. Torigoe 2016 [35] meanwhile reported that LBM levels decreased by 18 ± 8 g in the CHD group and increased by 20 ± 9 g in the EHD group.

### Protein and carbohydrate intake, water soluble vitamin levels and dry lean mass

Only one of the included studies provided results involving protein and carbohydrate intake: Schorr 2011 [37]. This study compared an EHD group to a CHD group. The results showed that within- and between-group differences were not significantly different after six months of follow up for protein intake (EHD group baseline: 1.01 g/kg/day [IQR 0.82–1.19] to six months; 1.08 g/kg/day [IQR 0.79–1.45]; CHD group baseline: 0.87 g/kg/day [IQR 0.57–1.31] to six months; 0.93 g/kg/day [IQR 0.68–1.66]) or carbohydrate intake (EHD group: baseline; 197.4 g/day [IQR 168–293.5] to six months; 232.9 g/day [IQR 169.0–328.4]; CHD group: baseline 163.4 g/day [IQR 133.4–312.9] to six months; 188.8 g/day [154.1–290.4]).

Spanner 2003 [34] reported water soluble vitamin levels as one of their outcomes. The authors analysed water-soluble vitamin levels for three groups. One group delivered EHD nocturnally, another delivered EHD via a short-daily method while the final group was CHD control. It is not reported when during the study period vitamin C levels were measured and raw data has not been provided. Overall, the authors stated that vitamin levels remained in the reference ranges for all three groups except for the nocturnal group, where “half” of the patients were deficient, with vitamin C levels of less than 0.2 mg/dl. Both the short-daily and nocturnal groups showed reduced levels of homocysteine compared to the CHD group (short-daily group mean homocysteine: 2.16 mg/L; nocturnal group mean homocysteine: 1.59 g/L; CHD mean homocysteine level: 2.61 mg/L). It was reported that the EHD group had a significantly reduced level of homocysteine (*P* = 0.022) compared with the CHD group.

With regard to dry lean mass, the results from Demirci 2013 [36] showed that there were no significant differences between an EHD and CHD group after 12 months of follow up (EHD group baseline: 12.2 ± 4.1 kg to 12 months; 12.7 ± 4.1 kg; CHD group baseline: 11.5 ± 3.9 kg to 12 months; 11.4 ± 3.9 kg).

## Discussion

The aim of this systematic review was to assess the impact of EHD on nutritional parameters, when compared to CHD. We identified five studies which met our eligibility criteria; one RCT [37] and four observational studies [33–36]. These studies reported on several of our pre-specified outcomes: BMI [33, 34, 37], LBM [33–35], water-soluble vitamin levels [34], dry lean mass [36], protein and energy intake [37].

The main findings of this review are that based on limited evidence EHD appears to have no effect on the nutritional parameters included, except for vitamin C and homocysteine levels. However, the data for vitamin C and homocysteine are from one study [35], and there is considerable uncertainty surrounding the results as the included studies were rated as being at serious/high risk of bias [33–35, 37] or moderate risk of bias [36]. For four of our main outcomes (BMI, LBM, protein, and energy intake), no meaningful clinical or statistical differences were shown between extended and conventional hemodialysis. While EHD was not shown to improve the above nutritional parameters, it conversely also did not impact them negatively. One study [34] suggested that EHD may reduce vitamin C levels, which is clinically important as this may lead to increased levels of inflammation amongst the hemodialysis population [41].

A previous systematic review was conducted in 2016 [15] which sought to assess the effect of EHD prescribed as nocturnal dialysis on nutritional status. They included all study designs in their systematic review if at least one of the nutritional outcomes they were searching for were included. This allowed them to include a greater number of studies than those included in this current systematic review. The previous review concluded that EHD is associated with significantly higher protein and energy intake which conflicts with the findings of this review. The difference between their findings and those of this review are likely explained by the inclusion of only studies with comparator groups in the current review. The inclusion of comparator groups partially reduces regression to the mean effects which could explain the findings of the previous review. Although similarly to our findings, the previous review reported that there is limited evidence to assess the effects of EHD on body composition and BMI. It is important that the effect of EHD on BMI is elucidated as this outcome is associated with mortality in the hemodialysis population [42].

The results from this systematic review (based on limited evidence) suggest that EHD solutions are unlikely to have detrimental effects on the nutritional status of hemodialysis patients. There is very weak evidence from two studies [35, 36] showing that EHD was associated with an increase in lean body and dry lean mass, however more robust evidence from a RCT is needed to test this hypothesis.

Clinicians should note that the results of this systematic review and a separate cross-sectional study [43] both indicated that extended hemodialysis has the potential to cause vitamin-C deficiency and so we advise that vitamin levels should be monitored in EHD patients wherever possible. The authors note that the KDOQI guidance [44] recommends vitamin supplementation if deficiency in vitamin levels is identified amongst hemodialysis patients.

Future research is required to elucidate the effects of EHD modalities on nutritional parameters. Table 3 outlines several implications for future clinical research. Generally, poor reporting was a major feature of the included studies. Future research should adhere to the Consolidated Standards of Reporting Trials (CONSORT) guidance [45]. Numerous sources of bias existed in the evidence, namely, bias due to lack of randomization, missing outcome data, small sample sizes and underpowered studies, lack of blinding of outcome assessors and baseline imbalances. These issues must be resolved in the future to produce reliable results that can be used to make clinical recommendations.

**Table 3:** Main Methodological Concerns Regarding The Included Studies

Aim	Methodological limitation	Rationale for inclusion
Improved reporting of trials	Lack of published study protocols	3/5 included studies did not report a study protocol in their full paper, nor did they provide guidance regarding how to access one otherwise [33, 34, 36] 2/5 included studies stated in their full text that a study protocol was approved by an ethics committee, but did not provide guidance on how to access these study protocols [35, 37] The lack of easily accessible study protocols meant that all included studies were graded to be at “moderate” risk of bias in selection of the reported result
	Poor reporting of statistical analysis	2/5 included studies did not provide information regarding statistical handling of confounding factors [34, 35] 3/5 included studies did not provide information regarding how they determined their sample size [33–35] All the included studies did not report adequate evidence regarding the statistical handling of missing outcome data to avoid bias [33–37]
	Poor reporting of missing outcome data	One study provided no information at all regarding participant drop out [35] Two studies only revealed participant drop out when viewing the results section, with no reasons provided for the missing outcome data [34, 37]
	Poor reporting of results	One study reported their results for LBM only in the form of a graph, and utilised inappropriate units (change in grams), making the results difficult to interpret [35]
Reduction of bias within trials	Bias due to missing outcome data	The mean percentage of participant dropout in the intervention groups of the included studies was 51.3% (range: 45–53.9%) Meanwhile, the mean percentage of participant dropout in the control groups of the included studies was 27.8% (range: 9.5–52.4%) Only one study [37] reported the use of intention-to-treat analysis, however, this was not used for their outcomes regarding dietary intake of proteins and carbohydrates
	Small sample sizes and underpowered studies	None of the included studies reported conducting an a priori power calculation to decide their sample sizes
	Lack of randomisation	Only one of the included studies was a randomised control trial [37]
	Lack of blinding outcome assessors	Only one of the included studies [33] reported blinding an outcome assessor for one of their included outcomes
	Baseline imbalances	One study [35] reported large differences between their control and intervention groups with regard to BMI, dry weight, and age, which may have impacted the results

The studies included in this systematic review are all dated, with the most recent one being 6 years old. Considering the potential beneficial effects of EHD on nutritional parameters and the lack of recent research, there is a crucial need for RCT data on the effect of these modalities on nutritional outcomes. For future randomised controlled studies in this area, we recommend reviewing the KDOQI guidance [44] and selecting those nutritional outcomes which are associated with mortality and are assessed as part of usual care to minimize missing outcome data. Authors may wish to assess differences in water soluble vitamin levels between CHD/EHD patients, and other markers of nutrition (i.e. protein and energy intake, LBM, circulating markers of appetite).

Despite our broad search strategy (i.e. the inclusion of non-randomised study designs), we were only able to retrieve five studies to include in this systematic review. Furthermore, whilst our aim was to conduct a meta-analysis (including sub-group and sensitivity analysis), this was not possible due to significant heterogeneity between the studies, and therefore a narrative synthesis was conducted. We did not assess for publication bias statistically using funnel plots (as stated in our study protocol). Despite this, the likelihood of publication bias is low as the included studies reported results which were not statistically significant.

The quality of the results of this systematic review are limited by the low quality of the included studies, as 4/5 studies were graded to be at serious risk of bias, and GRADE certainty assessment revealed low or very low certainty in the evidence. Another limitation of this study which is important to recognise is that two of the included studies had short follow-up periods, consisting of 2 months [35] and 6 months [37]. There was significant variability in the included studies with regard to the EHD regimens (long nocturnal, short daily) and the location where treatment was delivered (standard treatment was only in-centre, while extended treatment was delivered with a combination of in-centre and at home dialysis depending on the study). It is possible that these variations in EHD treatment could impact nutritional parameters differently, although it is unlikely that these differences would change the conclusions of this review. Finally, the average age of the participants in the EHD cohorts was younger than the average age of the participants in the CHD, which is likely attributed to selection bias within the studies.

### Practical application

Based on limited evidence, there is no suggestion that EHD has detrimental effects on the nutritional status of hemodialysis patients. We also recommend that where possible, clinicians monitor the water-soluble vitamin levels of hemodialysis patients and replace where appropriate. In the future, high-quality studies are required to elucidate what effect EHD has on nutritional parameters, so that clinical recommendations can be made.

## Appendix

See Table 4.

**Table 4:** Full search strategy for the MEDLINE database

	Search term
1	Dialysis
2	Hemodialysis
3	Haemodialysis
4	Renal dialysis
5	Renal replacement therapy
6	RRT
7	Kidney dialysis
8	Hemodiafiltration
9	Haemodiafiltration
10	1 OR 2 OR 3 OR 4 OR 5 OR 6 OR 7 OR 8 OR 9
11	Nocturnal
12	Nightly
13	Overnight
14	Night time
15	Extended
16	Frequent
17	Daily
18	Quotidian
19	11 OR 12 OR 13 OR 14 OR 15 OR 16 OR 17
20	Nutrition
21	Nutrition assessment
22	Malnutrition
23	Protein-energy malnutrition
24	Nutritional physiological phenomena
25	Body weight and measures
26	Food
27	Body composition
28	Energy
29	Proteins
30	Protein
31	Subjective global assessment
32	Nutritional status
33	Protein energy wasting
34	20 OR 21 OR 22 OR 23 OR 24 OR 25 OR 26 OR 27 OR 28 OR 29 OR 30 OR 31 OR 32 OR 33
35	10 AND 19 AND 34

## Abbreviations

BMI: Body Mass Index
CHD: Conventional Hemodialysis
CONSORT: Consolidated Standards of Reporting Trails
EHD: Extended Hemodialysis
ESKD: End Stage Kidney Disease
GRADE: Grading of Recommendations, Assessment, Development and Evaluations
IQR: Interquartile Range
LBM: Lean Body Mass
PEW: Protein Energy Wasting
RCT: Randomised Controlled Trial
SGA: Subjective Global Assessment
